# Fruit Pomace as a Functional Ingredient in Wheat Biscuits: Mineral Composition, Colour Characteristics, and Quality Evaluation of Peach Pomace-Enriched Biscuits

**DOI:** 10.3390/molecules31152633

**Published:** 2026-07-29

**Authors:** Sina Cosmulescu, Maria Bianca Mandache

**Affiliations:** 1 Department of Horticulture and Food Science, Faculty of Horticulture, University of Craiova, A.I. Cuza Street, No. 13, 200585 Craiova, Romania; 2 Doctoral School of Plant and Animal Resources Engineering, Faculty of Horticulture, University of Craiova, A.I. Cuza Street, No. 13, 200585 Craiova, Romania

**Keywords:** bakery fortification, nutritional profile, consumer acceptance, sustainable ingredients

## Abstract

Fruit pomace represents a valuable by-product of the fruit processing industry that can be reutilized to improve the nutritional quality of bakery products while contributing to sustainable waste valorisation. This study evaluated the effect of partially replacing wheat flour with apple, sour cherry, and peach pomace (5–15%) on the mineral composition and colour characteristics of biscuits and performed a detailed physicochemical and sensory assessment of biscuits enriched with peach pomace. Biscuits were prepared by substituting wheat flour with 5%, 10%, and 15% fruit pomace. Mineral composition and colour parameters were determined for biscuits containing all three pomace types. Based on the initial screening, peach pomace was selected for further physicochemical and sensory analyses. Fruit pomace significantly improved the mineral composition of biscuits. Sour cherry pomace increased Ca, Cu, and Mg contents, whereas peach pomace enhanced Fe, K, Mg, and Zn levels. Pomace addition also affected colour, resulting in lower lightness (L*) and higher redness (a*) and yellowness (b*), depending on pomace type and concentration. In peach pomace biscuits, increasing the substitution level led to higher moisture and acidity, while biscuit diameter and thickness decreased because of gluten network dilution. Sensory evaluation showed that biscuits containing 5–10% peach pomace achieved the highest scores for colour, aroma, taste, and overall acceptability. Fruit pomace can be successfully incorporated into wheat biscuits to improve their mineral composition while maintaining satisfactory quality attributes at appropriate substitution levels. The applied two-step approach identified peach pomace as the most suitable ingredient for biscuit fortification and supports the sustainable valorisation of fruit processing by-products in bakery applications.

## 1. Introduction

The food processing industry, particularly the fruit and vegetable sector, generates substantial amounts of by-products such as peels, pulp, and seeds. If not properly managed, these residues can pose environmental concerns due to their high moisture content, which promotes microbial growth and rapid degradation [1,2]. Fruit pomace, the main by-product of juice processing, is nevertheless a valuable source of bioactive compounds, including dietary fiber, minerals, proteins, and phenolic compounds [3]. Therefore, its sustainable valorisation represents an effective approach to reduce environmental impact while enhancing the economic efficiency of the agri-food sector [4,5]. In recent years, growing consumer awareness of the link between diet and health has led to a higher demand for foods enriched with natural bioactive compounds. This shift has stimulated the development of functional foods with potential health-promoting effects, reinforcing the concept of “food as medicine” [6,7,8]. In this context, fruit pomace has attracted considerable attention as a functional ingredient due to its nutritional profile and availability as a low-cost raw material. One of the most widely explored strategies involves the partial substitution of wheat flour with ingredients rich in fiber, minerals, and bioactive compounds [9]. Fruit pomace has been successfully incorporated into bakery products to improve their nutritional value, particularly by increasing dietary fiber and mineral content [10,11]. Among bakery products, biscuits represent a suitable matrix for fortification due to their widespread consumption, long shelf life, and technological flexibility [12]. Previous studies have demonstrated that the incorporation of fruit pomace can significantly enhance the nutritional profile of biscuits, contributing to the development of value-added functional products [13,14]. However, the incorporation of fruit pomace does not only affect nutritional composition, but also influences the technological and sensory properties of the final product. Due to their high fiber and pectin content, pomace powders can alter dough rheology by increasing water absorption capacity and interfering with gluten network development, leading to changes in texture, volume, and structural properties of baked products [15,16]. These modifications often result in denser products with reduced volume and increased hardness compared to conventional wheat-based products. In addition, natural pigments and phenolic compounds present in pomace can influence the colour of biscuits, generally leading to darker products as the level of incorporation increases [17]. Thermal processes such as Maillard reactions and caramelization may further intensify these colour changes [18,19]. From a sensory perspective, pomace addition may affect aroma, taste, texture, and overall acceptability. Previous studies have reported that moderate levels of pomace incorporation (e.g., 10–20%) can maintain acceptable sensory characteristics, while higher levels may negatively impact consumer perception [20]. Therefore, optimizing the incorporation level is essential to balance nutritional enhancement with product quality and consumer acceptance. Although fruit pomace has been widely investigated as an ingredient in bakery products, studies focusing on the effects of individual pomace types on the mineral composition and color characteristics of fortified biscuits remain limited. In addition, comprehensive evaluations of physicochemical and sensory properties are often restricted to selected formulations, leaving gaps in understanding the relationship between pomace type, product quality, and consumer acceptability. In this context, the study was based on the hypothesis that incorporating fruit pomace enhances the mineral content of wheat biscuits, with the extent of enrichment depending on the type and level of addition, while also influencing the product’s quality attributes. Accordingly, the aim was to evaluate the mineral composition and colour characteristics of biscuits enriched with apple, sour cherry, and peach pomace, and to perform a detailed physicochemical and sensory assessment of those formulated with peach pomace. A two-step experimental approach was applied, in which all pomace types were initially screened, and the most promising variant was subsequently selected for in-depth analysis. This approach was intended to identify the fruit pomace with the greatest potential for mineral enrichment while maintaining acceptable quality characteristics, thereby providing a scientific basis for the sustainable valorisation of fruit processing by-products and their use as ingredients in bakery products.

## 2. Results and Discussion

### 2.1. Mineral Element Content in Biscuits with Added Pomace

Given that fruit pomace represents a valuable natural source of minerals [21], its incorporation into white wheat flour biscuits may provide an effective strategy for developing functional foods with enhanced mineral intake. The data presented in Table 1 illustrate the influence of apple, sour cherry, and peach pomace additions (5, 10, and 15%) on the mineral composition of fortified biscuits. The analysed minerals included Ca (calcium), Cr (chromium), Cu (copper), Fe (iron), K (potassium), Mg (magnesium), Mn (manganese), Na (sodium), and Zn (zinc). Their concentrations varied significantly (*p* < 0.05) depending on both the type and level of pomace incorporated. Calcium content was differently affected by the pomace type. Biscuits fortified with sour cherry pomace (5–15%) showed a gradual increase from 14.68 to 19.67 mg/100 g, whereas samples with apple pomace displayed a plateau after the 10% addition (15.54 mg/100 g). In contrast, peach pomace formulations reached the highest calcium level at 5% addition (18.62 mg/100 g) and the lowest at 10% (17.37 mg/100 g). This non-linear trend may be attributed to matrix interactions affecting mineral availability, such as binding of calcium to fiber and other components at higher inclusion levels, as well as natural variability in raw material composition. Although the control sample showed the lowest calcium concentration (14.66 mg/100 g), it did not differ significantly from biscuits containing 5% sour cherry pomace. The values obtained for the control sample were consistent with those previously reported by the same authors [22], confirming the reproducibility of the experimental conditions. Overall, formulations enriched with sour cherry pomace exhibited higher calcium levels compared with those containing apple or peach pomace. This increase may be related to the naturally high calcium content of fruit pomace and possible interactions with phenolic compounds that enhance mineral retention during baking, thereby improving the nutritional profile of biscuits, as calcium plays a key role in bone health and metabolic processes [22].

Potassium concentration (Table 1) also increased with the pomace level. The lowest value was recorded in the control sample (66.60 mg/100 g), while biscuits containing 15% pomace showed higher contents (93.57, 106.77, and 129.65 mg/100 g), which can be attributed to the naturally high mineral content of fruit pomace [23]. A similar trend was observed for magnesium. Biscuits containing sour cherry and peach pomace exhibited the lowest magnesium levels at 5% addition (10.56 and 10.83 mg/100 g) and the highest at 15% (12.63 and 12.34 mg/100 g). In contrast, biscuits fortified with apple pomace showed a gradual decrease in magnesium content, from 10.74 mg/100 g at 5% to 9.95 mg/100 g at 15%, resulting in a lower concentration than that of the control sample (10.27 mg/100 g). Regarding sodium content (Table 1), the highest values were observed in biscuits containing 10% apple pomace (90.84 mg/100 g), 10% sour cherry pomace (64.35 mg/100 g), and 15% peach pomace (84.16 mg/100 g). The variations in sodium levels among samples may be associated with technological factors, particularly the non-uniform distribution of salt within the dough. During kneading, uneven dispersion of salt can lead to differences in sodium concentration [24]. In addition, variations in moisture content may influence ion distribution and sodium retention in the final product [25], while interactions between sodium ions and the protein network can further affect retention depending on the hydration degree and gluten structure [26]. However, in the present study, these effects were not statistically significant (*p* > 0.05), suggesting that the observed differences may be due to natural variability rather than a consistent treatment effect. Although the incorporation of fruit pomace increased the levels of calcium, magnesium, and potassium, the obtained values (Ca: 14.66–19.67 mg/100 g; Mg: 9.95–12.63 mg/100 g; K: 66.60–129.65 mg/100 g) represent only a small fraction of the recommended daily intake (NRV: Ca 800 mg, Mg 375 mg, K 2000 mg), corresponding to less than 15% per 100 g product, and therefore do not meet the criteria for nutritional claims such as “source of” these minerals. Table 2 presents the concentrations of the trace elements Cr, Cu, Fe, Mn, and Zn in the biscuit samples.

**Figure 1:**
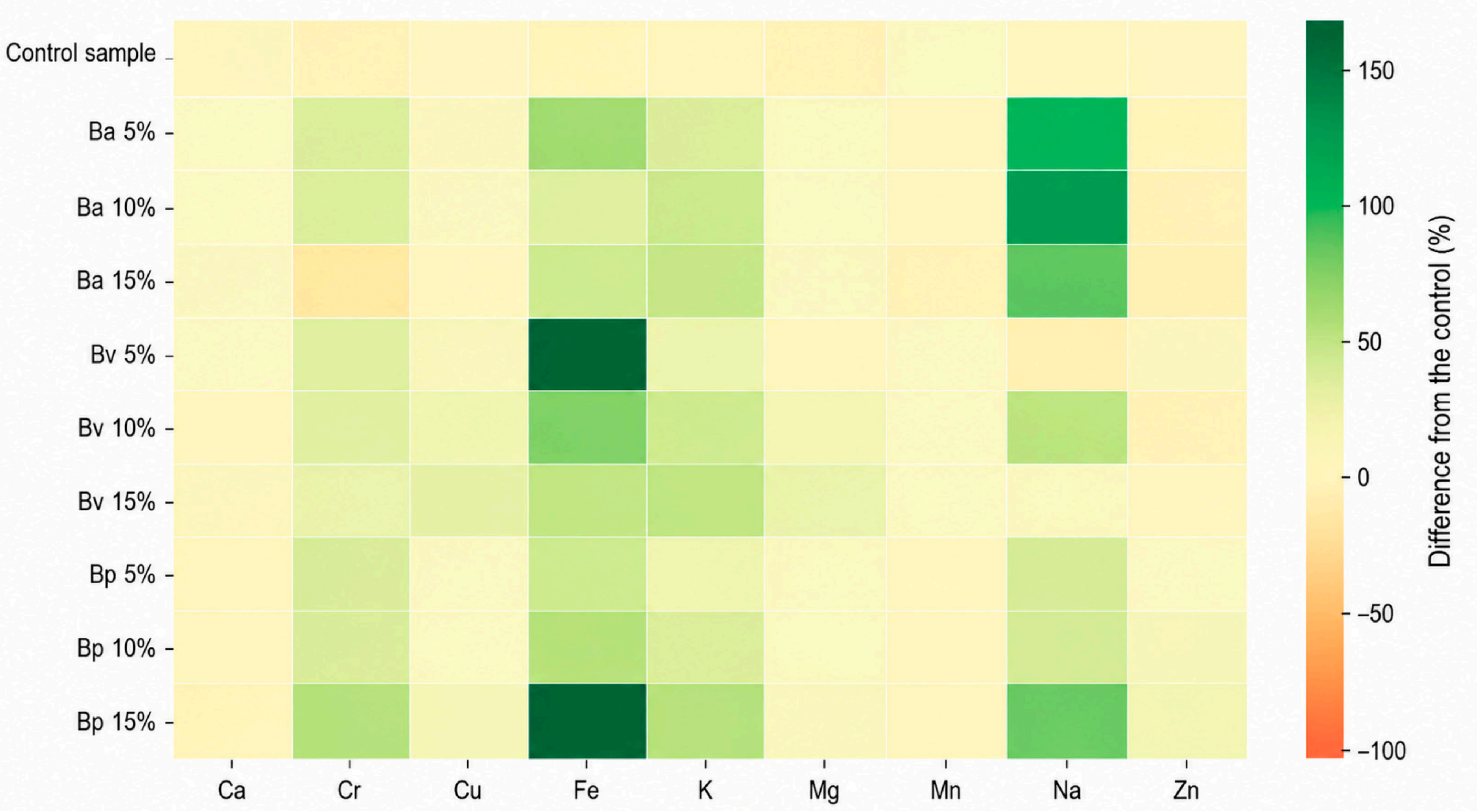
Summary of percentage differences with respect to the control.

Chromium concentrations ranged from 0.009 to 0.012 mg/100 g, with no marked differences among the control and pomace-enriched biscuits. The control sample contained 0.0098 mg/100 g Cr, while biscuits enriched with apple and sour cherry pomace exhibited similar values (0.009–0.011 mg/100 g). In biscuits containing peach pomace, chromium content showed a slight increase with increasing pomace addition, reaching 0.012 mg/100 g at the 15% substitution level. These results are consistent with the low chromium concentrations previously reported for the fruit pomaces [27] and indicate that the partial replacement of wheat flour with fruit pomace had only a limited effect on the chromium content of the final products. Copper content generally increased following partial substitution of wheat flour with fruit pomace compared with the control (0.11 mg/100 g). Biscuits containing sour cherry and peach pomace showed progressive increases (0.12–0.16 mg/100 g and 0.12–0.13 mg/100 g, respectively), whereas those fortified with apple pomace showed a slight decrease (0.12–0.11 mg/100 g). The highest copper concentrations were therefore recorded in sour cherry pomace biscuits. Iron content was strongly influenced by both pomace type and concentration. The control sample showed the lowest value (0.18 mg/100 g). In biscuits enriched with peach pomace, iron increased markedly (0.27–0.49 mg/100 g), while samples containing apple and sour cherry pomace showed gradual decreases (0.29–0.21 mg/100 g and 0.44–0.28 mg/100 g, respectively). Consequently, peach pomace formulations exhibited the highest iron concentrations, likely reflecting the intrinsic mineral content of the fruit and its retention during baking. Manganese levels showed moderate variations with pomace addition. Biscuits with apple pomace presented a gradual decrease, reaching the lowest value at 15% addition (0.19 mg/100 g), whereas sour cherry pomace formulations showed a slight increase (0.20–0.22 mg/100 g). A similar increase was observed in peach pomace biscuits, with the highest level also recorded at 15% addition (0.22 mg/100 g). Zinc content varied depending on pomace type. Compared with the control (0.38 mg/100 g), biscuits containing apple and sour cherry pomace exhibited slightly lower values (0.33–0.36 mg/100 g and 0.35–0.38 mg/100 g). In contrast, peach pomace significantly increased zinc concentration (0.38–0.55 mg/100 g). Comparable results were reported by Ivanišová et al. [28], who observed higher levels of minerals—particularly copper, zinc, manganese, and iron—in biscuits enriched with grape pomace compared with control samples. Overall, the mineral composition varied depending on the type and level of fruit pomace incorporated, resulting in increases, decreases, or relatively stable concentrations across the analysed biscuit samples (Figure 1).

The heatmap illustrating the percentage differences relative to the control sample highlights the overall trends in mineral variation. Notable increases were observed for iron, potassium, sodium, and zinc, particularly in biscuits containing higher levels of peach pomace (Bp 15%), while calcium, copper, magnesium, and manganese showed only minor changes. Chromium concentrations remained relatively stable across all formulations, exhibiting only slight variations compared with the control, with a modest increase in biscuits containing peach pomace. The color-coded heatmap provides a rapid visual overview of the minerals positively or negatively affected by fruit pomace incorporation, facilitating the interpretation of compositional changes in the fortified biscuits. Previous studies have reported variable effects of fruit pomace incorporation on the mineral composition of bakery products. In breads fortified with apple, sour cherry, and peach pomace, the levels of copper, iron, and potassium increased proportionally with pomace addition, whereas magnesium, calcium, chromium, sodium, and zinc showed irregular variations depending on pomace concentration [29]. Similarly, Andrejko et al. [30] reported that the addition of 10% apple pomace to biscuits reduced zinc and magnesium levels, while manganese and iron remained comparable to the control sample. In contrast, Mir et al. [31] observed a progressive increase in mineral elements (Ca, Cu, Fe, K, Mg, Na, Zn) with increasing apple pomace concentrations (3–9%). Comparable trends were reported by Filipović et al. [32], who found increased concentrations of K (127.73–185.49 mg/100 g), Ca (18.27–24.61 mg/100 g), Mg (25.43–33.37 mg/100 g), and Fe (1.31–1.41 mg/100 g) in biscuits supplemented with 15% dehydrated peach. Differences between mineral concentrations reported in the literature may be attributed to factors such as plant species, soil characteristics, climatic conditions, and agricultural practices, which influence mineral uptake and accumulation in plant materials [33]. Overall, the results highlight the distinct behaviour of mineral elements in fortified products, as each type of pomace interacts differently with the food matrix due to its specific mineral composition. Lidiková et al. [34] also reported that while minerals such as calcium, potassium, and magnesium may increase following pomace incorporation, other elements do not show a uniform trend. Furthermore, heat treatments applied during baking can modify nutrient composition and metal–matrix interactions, altering both the concentration and chemical forms of minerals [35,36]. In bakery systems, mineral retention and bioavailability depend not only on the initial mineral content but also on interactions between mineral ions and matrix components such as proteins, carbohydrates, and dietary fibers. Minerals may form complexes with proteins, while polysaccharides and fibers can adsorb or chelate metal ions, limiting their mobility and retention in the final product and influencing their solubility and bioavailability [37]. In addition, technological factors such as pH, moisture, and baking temperature can further affect mineral distribution and retention [38].

### 2.2. The Effect of the Addition of Pomace on the Colour of Biscuits

**Figure 2:**
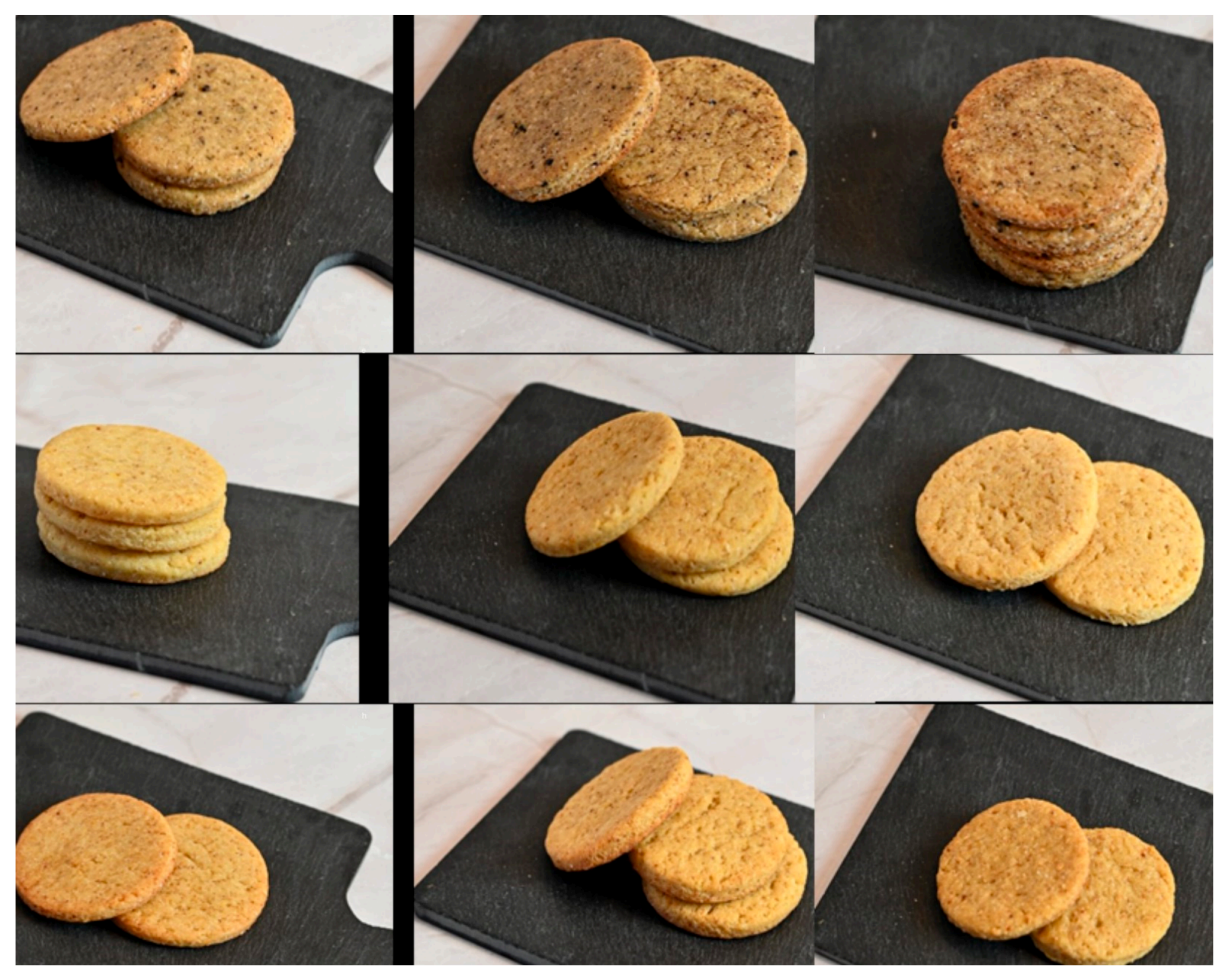
Representative images of biscuits enriched with sour cherry (**top** row), apple (**middle** row), and peach (**bottom** row) pomace at 5%, 10%, and 15% substitution levels (from left to right).

Colour is one of the essential parameters of the sensory characteristics of food products, significantly influencing their acceptability by consumers [39]. Analysis of the colorimetric parameters L* (lightness), a* and b* of biscuits fortified with different types and levels of pomace (5, 10 and 15%) highlights statistically significant differences (*p* < 0.05), as shown in Table 3 and Figure 2. Compared to the control sample (L* = 62.73), biscuits with apple pomace addition showed a progressive decrease in luminance with increasing substitution level, L* values varying between 60.70 and 44.22 for the range 5–15%. The sample with the highest addition level (15%) was characterized by the lowest luminance, a phenomenon explained by the interaction between reducing sugars [40] and phenolic compounds, an interaction that favours the development of the Maillard reaction and leads to the intensification of browning processes and the darkening of the product colour [15]. At the same time, the increase in the proportion of apple pomace determined a progressive increase in the a* parameter, from 5.97 in the control sample to 14.39 in the sample with 15% addition. The b* parameter also recorded an increase compared to the control sample (17.80), the values varying between 20.69 and 30.10 depending on the concentration of pomace used.

A similar behaviour in terms of luminance reduction was also observed in the case of biscuits with cherry pomace. With the increase in the addition level (5–15%), the L* values decreased from 51.30 to 36.02, indicating an intensification of the colour of the products. In contrast, the chromaticity a* increased progressively (10.06–16.93), while the values of the parameter b* recorded a slight reduction (27.72–22.85). The amplification of the a* values can be associated with the oxidation of the pomace polyphenols, which represent a substrate for enzymatic browning reactions [41]. In general, enzymatic browning contributes to a greater extent to the formation of red and yellow shades than non-enzymatic browning processes [42]. Also, the higher values of the a* parameter observed in the case of cherry marc biscuits can be attributed to the increased anthocyanin content characteristic of this type of raw material. In the case of biscuits fortified with peach pomace, increasing the addition level (5–15%) also caused a significant reduction in luminance, with L* values ranging between 48.14 and 41.39. At the same time, the a* and b* parameters showed an increasing trend, with a* values ranging between 11.06 and 15.84, and those of the b* parameter between 25.57 and 30.95. The total colour difference (ΔE) of the biscuits increased proportionally with the added peach pomace level, reaching maximum values ranging between 23.76 and 29.65 at the 15% substitution level. Colour changes were observed even at the lowest addition level, with ΔE values ranging between 3.96 and 17.29, indicating a visible change in the chromatic parameters of the final products. These variations can be explained both by the presence of phenolic compounds, including anthocyanins, and by the influence of technological processes that can intensify or modify the colour of formulated products. Similar results were also reported by Mir et al. [31], who highlighted the effect of adding apple pomace on the properties of biscuits. Therefore, fruit pomace can be considered not only a valuable source of bioactive compounds, but also a supplier of natural pigments that contribute to improving the functional and visual properties of food products.

### 2.3. Physico-Chemical Characteristics of Biscuits with Added Fruit Pomace

The physicochemical characteristics of biscuits formulated with peach pomace (Pp), at substitution levels of 5–15%, are presented in Table 4. The properties of biscuits with apple and sour cherry pomace have been reported in previous studies by the same authors and are not detailed in the present work, which focuses on the evaluation of biscuits with peach pomace [22].

The results show a very small increase in moisture content with increasing proportion of peach pomace. The control sample presented a moisture content of 6.83%, while the biscuits with 15% addition recorded a value of 7.22%. Similar trends were reported by Usman et al. [43], who observed an increase in the moisture content of biscuits supplemented with apple pomace, as well as by Shabnam et al. [44] and Naseem et al. [41] for biscuits enriched with peach and apple pomace. The values obtained in this study remain below the threshold of 13%, the limit at which microbial growth is inhibited, suggesting a good potential for stability during storage [45]. The acidity of biscuits with peach pomace increased with the level of substitution. The control sample presented an acidity of 1.8 degrees, and biscuits with 15% pomace recorded a value of 5.2 degrees. These results are consistent with those reported by Tarasevičienė et al. [46], who highlighted the increase in acidity in bakery products fortified with pomace.

From a morphometric point of view, the increase in the substitution level caused the biscuit dimensions to be reduced compared to the control sample (height 10.65 mm; diameter 55.77 mm). The biscuits with peach pomace showed lower values of height (9.00–8.17 mm) and diameter (52.40–50.63 mm). These changes are attributed to the weakening of the gluten and starch network as a result of the addition of pomace fibres, a phenomenon also reported by Usman et al. [43] and De Toledo et al. [47]. The reduction in biscuit height can be explained by structural changes in the dough, since expansion during baking depends on the stability of the gluten network, leavening agents and thermally generated water vapor [48]. Partial replacement of wheat flour with fibre-rich ingredients, such as pomace, reduces gluten content and leads to products with lower volume and a more compact structure [32,49]. The diameter/height ratio, an important indicator of biscuit quality, was higher in all formulations with peach pomace compared to the control sample (5.24), reaching values up to 6.20 at the 15% substitution level. The same trend was observed in other studies, where increasing the pomace level led to an increase in this ratio [41,43]. In our previous work [22], apple and sour cherry pomace (5–15%) were successfully used to enrich biscuits, improving their content of bioactive compounds and antioxidant activity. These results demonstrated the potential of fruit pomace as a functional ingredient and provided the basis for the present study.

### 2.4. Evaluation of the Sensory Characteristics of Biscuits with the Addition of Peach Pomace

The results of the sensory evaluation indicate that the addition of peach pomace significantly influenced the organoleptic characteristics of the formulated biscuits, with scores varying depending on the level of substitution (Table 5). The sensory properties of biscuits enriched with apple and sour cherry pomace have been previously investigated by the same authors [22] and are not detailed in the present work, which focuses on the evaluation of biscuits with peach pomace.

The products obtained presented the typical characteristics of this assortment, being appreciated for their uniform, slightly glossy and well-browned surface. The internal structure of the biscuits was appropriate; they were well baked, with fine porosity and without obvious defects. The analysis of the sensory scores highlights that moderate levels of peach pomace (5–10%) generated the highest scores for most of the evaluated parameters. For the external aspect parameter, biscuits with 5% pomace obtained the highest score (8.67), being significantly superior to the control sample (8.17), while at the 15% level a significant decrease was recorded (7.50). These results confirm that a low addition of pomace does not negatively affect the acceptability of bakery products, an observation also reported by Kohajdová et al. [50]. Regarding consistency, all formulations with peach pomace presented lower values compared to the control sample (8.67), with scores of 8.00, 7.50 and 7.00 for the 5%, 10% and 15% levels, respectively. The decrease in consistency can be attributed to the structural changes in the dough determined by the presence of dietary fibres in pomace, a phenomenon also described by Dhingra et al. [51].

For the colour parameter, biscuits with 5% and 10% peach pomace obtained the highest scores (9.00), being significantly superior to the control sample (7.00), while at the 15% level the score was slightly lower (8.83). The improvement in colour can be associated with the presence of natural pigments and phenolic compounds in the pomace, which contribute to the intensification of the product colour, as also reported by Andrés-Bello et al. [52]. This is consistent with the instrumental colour data (CIE L*, a*, b*), which showed darker and more reddish tones, in agreement with the sensory evaluation where panelists perceived a more intense and appealing colour. A similar behaviour was observed for smell and taste, where biscuits with 5% and 10% pomace recorded the highest scores (9.00 and 8.67), compared to the control sample (8.00), while at the 15% level a significant decrease was observed (7.33). These results are consistent with the observations reported by Kohajdová et al. [50] and O’Shea et al. [53], who highlighted the positive effect of the aromatic compounds in pomace on the sensory properties. In terms of overall acceptability, the best evaluated formulation was the one with 5% peach pomace (9.00), followed by the control sample and the biscuits with 10% pomace (8.33), while the 15% level led to a significant decrease in acceptability (7.50). These results confirm that moderate levels of substitution contribute to maintaining high acceptability, while higher levels may negatively affect consumer perception. Similar results were also reported by Salehi and Aghajanzadeh [49] and Shabnam et al. [44], who highlighted that the acceptability of cereal-based products enriched with fruit ingredients directly depends on the proportion of addition used. It has also been reported that at high levels of substitution (above 20%), the structure of the products can become more compact and less uniform [38], which may explain the decrease in sensory scores observed at the 15% level. It is found that the integration of peach pomace into biscuit formulations contributes to the improvement of sensory characteristics when used in moderate proportions. The 5% level has been shown to be optimal for obtaining a product with high sensory acceptability, while higher levels may negatively affect certain quality attributes.

## 3. Materials and Methods

### 3.1. Raw Materials

Apple (*Malus domestica*), sour cherry (*Prunus cerasus*), and peach (*Prunus persica*) pomace, obtained as by-products of cold-pressed juice production, were sourced from two local processors. The samples were dried in a laboratory oven at 50–60 °C until the moisture content decreased to around 10%. Subsequently, the dried pomace was ground and passed through a 0.55 mm sieve to obtain fine powders. The pomace powders were stored in hermetically sealed glass containers at 4 °C until further use. The mineral composition of apple, sour cherry, and peach pomace has been previously reported by the same authors [27] and is therefore not detailed in the present study.

### 3.2. Biscuit Formulation and Preparation

The formulation of the biscuits is presented in Table 6. Wheat biscuits were prepared in a confectionery laboratory using wheat flour type 000, eggs, milk powder, sunflower oil, sugar, iodized salt, and baking powder. Wheat flour was partially replaced with apple, sour cherry, and peach pomace powders at substitution levels of 5%, 10%, and 15% to evaluate mineral enrichment and colour characteristics. For the detailed physicochemical and sensory evaluation, only biscuits containing peach pomace were further analyzed.

All ingredients were mixed in a professional planetary mixer (type KitchenAid Professional 5 Plus, Greenville, OH, USA) at medium speed (level 2–3) for approximately 8–10 min until a homogeneous dough was obtained. The dough was then laminated and shaped using a stainless-steel mold. The biscuits were baked in a convection oven (professional laboratory oven, Memmert, Schwabach, Germany) at 180 °C for 10 min and then cooled at room temperature (21–22 °C). The biscuit samples were coded as follows: Ba—biscuits containing apple pomace; Bv—biscuits containing sour cherry pomace; Bp—biscuits containing peach pomace. The control biscuit formulation and processing conditions were identical to those previously reported by the same authors [27], ensuring comparability of the results.

### 3.3. Mineral Analysis of Tested Biscuits

The mineral composition of pomace-enriched biscuits (apple, sour cherry, and peach) was determined using inductively coupled plasma optical emission spectrometry (ICP-OES). Approximately 4.8–5.1 g of each biscuit sample was subjected to cold digestion with 15 mL of spectrally pure nitric acid (Merck-Sigma-Aldrich, Darmstadt, Germany) for six weeks. This extended digestion period was applied to ensure complete mineralization of the complex biscuit matrix, particularly rich in organic matter, in accordance with established procedures [22]. The digested solutions were filtered and diluted to a final volume of 25 mL with ultrapure water. Elemental concentrations (Ca, Cr, Cu, Fe, K, Mg, Mn, Na, and Zn) were quantified using an ICP-OES instrument (Thermo Scientific, Waltham, MA, USA, iCAP 6000), following the methodology described by Dróżdż et al. [54]. The instrumental operating conditions were: RF power 1.15 kW, auxiliary Ar gas flow rate 0.2 L min^−1^, nebulizer Ar gas flow rate 0.42 L min^−1^, and coolant gas flow rate 12 L min^−1^. The analytical wavelengths used were: Ca 422.673 nm, Cr 283.563 nm, Cu 224.700 nm, Fe 259.940 nm, K 766.490 nm, Mg 280.270 nm, Mn 257.610 nm, Na 589.592 nm, and Zn 213.856 nm.

### 3.4. Colour Measurement of Tested Biscuits

Colour parameters were determined using the CIE Lab* system with a reflectance colorimeter (PCE-CSM 2, PCE Instruments, Manchester, UK). The total colour difference (ΔE) was calculated as ΔE = [(L* − L_0_*)^2^ + (a* − a_0_*)^2^ + (b* − b_0_*)^2^]^1/2^, relative to the control sample. Values were interpreted as follows: ΔE < 1 (not perceptible), 1–3 (slightly perceptible), and >3 (clearly perceptible).

### 3.5. Physicochemical Analysis of Tested Biscuits

The physicochemical properties were determined for biscuits containing peach pomace, selected based on preliminary results as the most promising formulation. The evaluated parameters included moisture content, acidity, and dimensional characteristics (diameter, thickness, and diameter-to-thickness ratio). Moisture content was determined gravimetrically by drying samples to constant weight according to AOAC standard methods, while acidity was measured by titration following AACC standard procedures. Biscuit dimensions were measured using a digital caliper (electronic caliper, Mitutoyo, Kawasaki, Japan) [22,27].

### 3.6. Sensory Evaluation of Tested Biscuits

Sensory evaluation was conducted for biscuits containing peach pomace using a panel of ten semi-trained evaluators (4 males and 6 females) and a nine-point hedonic scale (1 = dislike extremely; 9 = like extremely). The evaluated attributes included appearance, colour, texture, aroma, taste, and overall acceptability. Samples were presented at room temperature in randomized, coded order, with water provided for palate cleansing. The evaluation followed standard guidelines (e.g., ISO 8589) [55], and informed consent was obtained from all participants.

### 3.7. Statistical Analysis

All analyses were performed in triplicate, and results were expressed as mean ± standard deviation. Statistical differences among samples were assessed using one-way analysis of variance (ANOVA), followed by Duncan’s multiple range test, with significance set at *p* < 0.05. All statistical analyses were conducted using IBM SPSS Statistics 26.

## 4. Conclusions

The present study demonstrates that apple, sour cherry, and peach pomace can be effectively used to fortify wheat biscuits, leading to measurable increases in mineral content, particularly potassium (66.60–129.65 mg/100 g), alongside moderate increases in calcium and magnesium. Pomace addition significantly affected colour parameters, resulting in decreased lightness (L*: 62.73–36.02) and increased redness (a*) and yellowness (b*), depending on the type and level of substitution. In peach pomace biscuits, the increase in moisture and acidity, together with reduced diameter and thickness, suggests formulation-related effects associated with gluten dilution and matrix interactions. Sensory evaluation identified 5–10% as the optimal incorporation level, while 15% negatively impacted acceptability. The sensory assessment was conducted using a semi-trained panel consisting of 10 participants. Overall, the findings support the use of fruit pomace as a functional ingredient to enhance mineral profile and promote the sustainable valorisation of agro-industrial by-products, while maintaining product quality.

## Figures and Tables

**Table 1:** The main mineral content of biscuits fortified with apple, cherry, and peach pomace *.

Sample *	Ca (mg/100 g)	K (mg/100 g)	Mg (mg/100 g)	Na (mg/100 g)
Control biscuits	14.66 ± 0.007 ^h^	66.60 ± 0.05 ^j^	10.27 ± 0.03 ^i^	43.43 ± 0.02 ^i^
Ba 5%	15.27 ± 0.07 ^g^	84.72 ± 0.26 ^h^	10.74 ± 0.04 ^f^	86.13 ± 0.32 ^b^
Ba 10%	15.54 ± 0.02 ^f^	92.61 ± 0.16 ^f^	10.40 ± 0.02 ^h^	90.84 ± 0.26 ^a^
Ba 15%	14.60 ± 0.01 ^i^	93.57 ± 0.07 ^e^	9.95 ± 0.02 ^j^	81.70 ± 0.15 ^d^
Bv 5%	14.68 ± 0.03 ^h^	77.43 ± 0.14 ^i^	10.56 ± 0.002 ^g^	38.17 ± 0.09 ^j^
Bv 10%	17.85 ± 0.01 ^d^	93.85 ± 0.07 ^d^	11.14 ± 0.006 ^d^	64.35 ± 0.19 ^e^
Bv 15%	19.67 ± 0.01 ^a^	106.77 ± 0.06 ^b^	12.63 ± 0.03 ^a^	45.78 ± 0.07 ^h^
Bp 5%	18.62 ± 0.05 ^b^	87.53 ± 0.28 ^g^	10.83 ± 0.06 ^e^	60.05 ± 0.11 ^f^
Bp 10%	17.37 ± 0.02 ^e^	105.89 ± 0.16 ^c^	11.31 ± 0.005 ^c^	59.78 ± 0.08 ^g^
Bp 15%	18.17 ± 0.01 ^c^	129.65 ± 0.04 ^a^	12.34 ± 0.03 ^b^	84.16 ± 0.14 ^c^

* Ba 5,10,15%-biscuits with the addition of 5,10,15% apple pomace; Bv 5,10,15%-biscuits with the addition of 5,10,15% cherry pomace; Bp 5,10,15%-biscuits with the addition of 5,10,15% peach pomace; X = mean; SD = standard deviation (*n* = 3). The distinct letters indicate statistically significant differences (Duncan test with multiple intervals, *p* < 0.05).

**Table 2:** The trace element content of biscuits fortified with apple, cherry, and peach pomace *.

Sample *	Cr (mg/100 g)	Cu (mg/100 g)	Fe (mg/100 g)	Mn (mg/100 g)	Zn (mg/100 g)
Control biscuits	0.0098 ± 0.0007 ^a^	0.11 ± 0.0001 ^c^	0.18 ± 0.00005 ^d^	0.21 ± 0.00004 ^a^	0.38 ± 0.0001 ^c^
Ba 5%	0.011 ± 0.0009 ^a^	0.12 ± 0.0001 ^bc^	0.29 ± 0.0009 ^c^	0.22 ± 0.0007 ^a^	0.36 ± 0.0005 ^cd^
Ba 10%	0.011 ± 0.0008 ^a^	0.12 ± 0.0002 ^bc^	0.20 ± 0.0005 ^d^	0.21 ± 0.0005 ^a^	0.35 ± 0.0007 ^cd^
Ba 15%	0.009 ± 0.0001 ^a^	0.11 ± 0.0001 ^c^	0.21 ± 0.0001 ^d^	0.19 ± 0.0001 ^a^	0.33 ± 0.0004 ^d^
Bv 5%	0.011 ± 0.0001 ^a^	0.12 ± 0.0004 ^bc^	0.44 ± 0.0007 ^b^	0.20 ± 0.0003 ^a^	0.38 ± 0.0008 ^c^
Bv 10%	0.011 ± 0.0006 ^a^	0.15 ± 0.0007 ^ab^	0.31 ± 0.0005 ^c^	0.20 ± 0.0003 ^a^	0.35 ± 0.0001 ^cd^
Bv 15%	0.010 ± 0.0006 ^a^	0.16 ± 0.0003 ^a^	0.28 ± 0.0001 ^c^	0.22 ± 0.0001 ^a^	0.36 ± 0.0006 ^cd^
Bp 5%	0.010 ± 0.0001 ^a^	0.12 ± 0.0002 ^bc^	0.27 ± 0.0008 ^c^	0.21 ± 0.0007 ^a^	0.38 ± 0.0008 ^c^
Bp 10%	0.011 ± 0.0007 ^a^	0.12 ± 0.0002 ^bc^	0.30 ± 0.0007 ^c^	0.20 ± 0.0004 ^a^	0.43 ± 0.0003 ^b^
Bp 15%	0.012 ± 0.0001 ^a^	0.13 ± 0.0001 ^abc^	0.49 ± 0.0001 ^a^	0.22 ± 0.0008 ^a^	0.55 ± 0.0003 ^a^

* Ba 5,10,15%-biscuits with the addition of 5,10,15% apple pomace; Bv 5,10,15%-biscuits with the addition of 5,10,15% sour cherry pomace; Bp 5,10,15%-biscuits with the addition of 5,10,15% peach pomace; X = mean; SD = standard deviation (*n* = 3). Separate letters indicate statistically significant differences (Duncan multiple range test, *p* < 0.05).

**Table 3:** Colour parameters of biscuits fortified with different fruit pomace at various substitution levels *.

Addition (%)	Pomace Type	L* (X ± SD)	a* (X ± SD)	b* (X ± SD)	ΔE
0	Control	62.73 ± 0.58 ^a^	5.97 ± 0.24 ^a^	17.80 ± 0.70 ^a^	–
5	Apple	60.70 ± 0.78 ^b^	7.77 ± 0.27 ^b^	20.69 ± 0.38 ^b^	3.96 ± 0.37
	Sour cherry	51.30 ± 1.87 ^c^	10.06 ± 0.65 ^c^	27.72 ± 1.19 ^c^	15.67 ± 1.43
	Peach	48.14 ± 2.30 ^d^	11.06 ± 1.39 ^cd^	25.57 ± 0.99 ^c^	17.29 ± 2.14
10	Apple	53.87 ± 1.35 ^c^	8.93 ± 0.40 ^bc^	22.37 ± 1.22 ^b^	10.39 ± 1.53
	Sour cherry	41.03 ± 1.67 ^e^	11.01 ± 1.35 ^cd^	23.20 ± 2.30 ^bc^	22.70 ± 2.22
	Peach	46.65 ± 1.25 ^d^	14.73 ± 0.59 ^de^	28.41 ± 0.82 ^cd^	18.56 ± 0.75
15	Apple	44.22 ± 1.83 ^de^	14.39 ± 2.02 ^de^	30.10 ± 0.35 ^d^	23.76 ± 2.20
	Sour cherry	36.02 ± 2.27 ^f^	16.93 ± 1.44 ^f^	22.85 ± 1.41 ^bc^	29.30 ± 2.18
	Peach	41.39 ± 1.84	15.84 ± 1.20 ^ef^	30.95 ± 0.60 ^d^	29.65 ± 1.58

* L*-lightness, a* (positive)-red; (negative)-green, b* (positive)-yellow; (negative)-blue; total color difference E. X = mean; SD = standard deviation; CV% = coefficient of variation (*n* = 6). Different superscript letters within the same column indicate statistically significant differences (*p* < 0.05), according to Duncan’s multiple range test.

**Table 4:** Physicochemical characteristics of formulated biscuits *.

Sample	Humidity (%)	Acidity (Degrees)	Height (mm)	Diameter (mm)	Diameter/Height Ratio
Control sample	6.83 ± 0.75 ^a^	1.80 ± 0.02 ^a^	10.65 ± 0.27 ^a^	55.77 ± 0.45 ^a^	5.24 ± 0.15 ^a^
BP 5%	7.16 ± 0.79 ^b^	4.30 ± 0.07 ^b^	9.00 ± 0.26 ^b^	52.40 ± 0.52 ^b^	5.82 ± 0.18 ^b^
BP 10%	7.19 ± 0.79 ^b^	4.90 ± 0.06 ^c^	8.47 ± 0.12 ^bc^	51.80 ± 0.95 ^bc^	6.12 ± 0.14 ^c^
BP 15%	7.22 ± 0.79 ^b^	5.20 ± 0.06 ^c^	8.17 ± 0.38 ^c^	50.63 ± 0.90 ^c^	6.20 ± 0.22 ^c^

* Values are expressed as mean ± standard deviation. Different letters within the same column indicate significant differences (*p* < 0.05) according to Duncan’s multiple range test.

**Table 5:** Sensory scores of biscuits fortified with pomace (hedonic scale 1–9) *.

Sample	Exterior Appearance	Consistency	Colour	Smell and Taste	Overall Acceptability
Control	8.17 ± 0.52 ^b^	8.67 ± 0.48 ^a^	7.00 ± 0.67 ^c^	8.00 ± 0.58 ^b^	8.33 ± 0.49 ^b^
BP 5%	8.67 ± 0.49 ^a^	8.00 ± 0.58 ^b^	9.00 ± 0.00 ^a^	9.00 ± 0.00 ^a^	9.00 ± 0.00 ^a^
BP 10%	8.17 ± 0.58 ^b^	7.50 ± 0.53 ^c^	9.00 ± 0.00 ^a^	8.67 ± 0.49 ^a^	8.33 ± 0.49 ^b^
BP 15%	7.50 ± 0.53 ^c^	7.00 ± 0.67 ^c^	8.83 ± 0.39 ^b^	7.33 ± 0.65 ^c^	7.50 ± 0.53 ^c^

* Values represent mean ± standard deviation (*n* = 10). Different superscript letters within the same column indicate significant differences (Duncan test, *p* < 0.05).

**Table 6:** Formulation of wheat biscuits with different levels of pomace substitution.

Sample	Wheat Flour (g)	Pomace (g)	Eggs (g)	Milk Powder (g)	Oil (mL)	Sugar (g)	Salt (g)	Baking Powder (g)
Control	500	0	168	50	166	213	2	2
5%	475	25	168	50	166	213	2	2
10%	450	50	168	50	166	213	2	2
15%	425	75	168	50	166	213	2	2

## Data Availability

The original contributions presented in this study are included in the article. Further inquiries can be directed to the corresponding authors.
